# Raman fingerprint of two terahertz spin wave branches in a two-dimensional honeycomb Ising ferromagnet

**DOI:** 10.1038/s41467-018-07547-6

**Published:** 2018-11-30

**Authors:** Wencan Jin, Hyun Ho Kim, Zhipeng Ye, Siwen Li, Pouyan Rezaie, Fabian Diaz, Saad Siddiq, Eric Wauer, Bowen Yang, Chenghe Li, Shangjie Tian, Kai Sun, Hechang Lei, Adam W. Tsen, Liuyan Zhao, Rui He

**Affiliations:** 1 Department of Physics, University of Michigan, 450 Church Street, Ann Arbor, Michigan 48109 USA; 2 Institute for Quantum Computing, Department of Chemistry, and Department of Physics and Astronomy, University of Waterloo, Waterloo, 200 University Ave W, Ontario, N2L 3G1 Canada; 3 Department of Electrical and Computer Engineering, Texas Tech University, 910 Boston Avenue, Lubbock, Texas 79409 USA; 4 Department of Physics and Beijing Key Laboratory of Opto-electronic Functional Materials & Micro-nano Devices, Renmin University of China, Beijing, 100872 China

## Abstract

Two-dimensional (2D) magnetism has been long sought-after and only very recently realized in atomic crystals of magnetic van der Waals materials. So far, a comprehensive understanding of the magnetic excitations in such 2D magnets remains missing. Here we report polarized micro-Raman spectroscopy studies on a 2D honeycomb ferromagnet CrI_3_. We show the definitive evidence of two sets of zero-momentum spin waves at frequencies of 2.28 terahertz (THz) and 3.75 THz, respectively, that are three orders of magnitude higher than those of conventional ferromagnets. By tracking the thickness dependence of both spin waves, we reveal that both are surface spin waves with lifetimes an order of magnitude longer than their temporal periods. Our results of two branches of high-frequency, long-lived surface spin waves in 2D CrI_3_ demonstrate intriguing spin dynamics and intricate interplay with fluctuations in the 2D limit, thus opening up opportunities for ultrafast spintronics incorporating 2D magnets.

## Abstract (web summary)

Characteristics of spin waves in recently discovered two-dimensional (2D) Ising ferromagnets are still lacking. Here, Jin and Kim et al. report Raman resonance evidence of two sets of surface spin waves in the 2D honeycomb ferromagnet CrI_3_.

## Introduction

The recent discovery of two-dimensional (2D) ferromagnetism^1,2^ proves that magnetic anisotropy can overcome thermal fluctuations and stabilize long-range magnetic orders in the 2D limit at finite temperatures. This has immediately triggered tremendous interest^3–12^ in potential 2D magnet-based applications, ranging from ultrathin magnetic sensors to high-efficiency spin filter devices. Naturally, a complete description of the 2D magnetic phase is now needed, which requires not only the identification of the ordered ground state, but equally important, the understanding of the excitations, i.e., spin waves, or equivalently, magnons^13,14^. To date, there have been no direct comprehensive experimental studies on the full characteristics of spin waves in these 2D Ising ferromagnets, aside from the quasiparticle excitation spectra from the inelastic tunneling measurements in ref. ^11^.

A spin wave describes the spin dynamics of magnetic ordering when excited, and its frequency determines the ultimate switching speed of state-of-the-art ultrafast spintronic devices^15–18^. Generally speaking, the well-established spintronic devices based on Heisenberg ferromagnets have speeds in the gigahertz (GHz) regime due to the weak magnetic anisotropy^19,20^, while the speeds of the recently proposed antiferromagnet-based spintronic devices fall into the terahertz (THz) range owing to the exchange interaction between the two sublattices of the antiferromagnets^21–23^. Remarkably, the newly discovered 2D Ising honeycomb ferromagnet CrI_3_ possesses both merits for realizing high-frequency spin waves: the strong magnetic anisotropy from the Ising-type spin interactions^24^ and the large exchange coupling between the two Cr^3+^ sublattices within the honeycomb framework^25^.

In this work, we study the spin wave excitations in the 2D Ising honeycomb ferromagnet CrI_3_ using polarized micro-Raman spectroscopy. Based on Raman symmetry analysis, we uniquely distinguish two spin wave modes at 2.28 and 3.75 THz from the rest of the optical phonon modes. The doubling of the spin wave mode number is a direct consequence of the underlying non-Bravais honeycomb lattice, while the exceptionally high THz frequency for a ferromagnet reflects the strong magnetic anisotropy and exchange interactions in good agreement with theoretical expectations. From the temperature and thickness dependence of the spin wave characteristics, we show that short-range magnetic correlations are set in prior to the formation of a long-range static magnetic order for every thickness, and that stronger fluctuation effects appear in thinner flakes. Remarkably, we found that the integrated intensities (I. I.) of the two spin wave modes exhibit nearly no thickness dependence, in stark contrast to the fact that the I. I. for all optical phonons scale linearly with their sample thickness, as expected for all bulk modes in quasi-2D-layered materials. This observation on the two spin waves, however, shows striking analogy to the surface modes whose I. I. is independent of the thickness. Moreover, we show that, from more than ten-layer to monolayer CrI_3_, the spin wave frequencies (2.28 and 3.75 THz) and the onset temperatures (45 K) remain nearly constant, while their lifetimes decrease significantly from 50 and 100 ps to 15 ps, but remain an order of magnitude longer than their corresponding spin wave temporal periods.

## Results

### Spin wave dispersion calculations in 2D CrI_3_

**Fig. 1:**
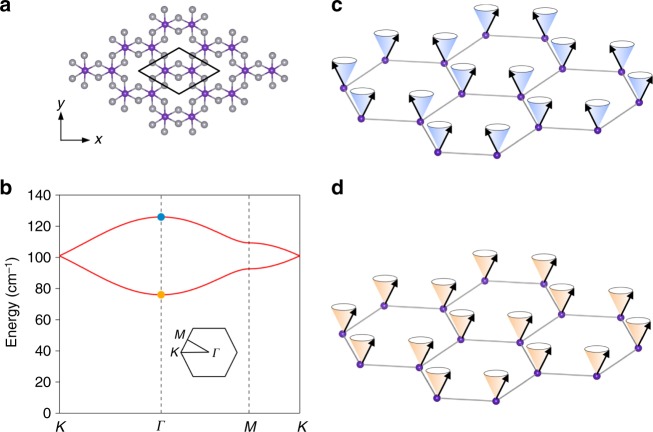
Magnetic excitations in the 2D Ising ferromagnet CrI_3_. **a** Top view of the atomic structure of monolayer CrI_3_, with Cr^3+^ in purple and I^–^ in gray. Cr^3+^ ions form a honeycomb lattice with two Cr^3+^ ions per unit cell (black enclosure). **b** Calculated magnon dispersion relation in monolayer CrI_3_. The inset shows the Brillouin zone. Magnon modes at the zone center (*Γ* point, zero-momentum) are highlighted by yellow and blue solid circles. **c**, **d** Schematic of the zone center magnon modes. The cones represent the precession trajectories of the spins (black arrows). The precession of spins on two Cr^3+^ sublattices are out-of-phase (**c**) and in-phase (**d**), which corresponds to the high- and low-energy modes, respectively, in **b**

We first performed the spin wave dispersion relation calculations for the monolayer ferromagnet CrI_3_, in which the Cr^3+^ cations form a honeycomb structure with the edge-sharing octahedral coordination formed by six I^−^ anions and the magnetic moment of the two Cr^3+^ cations (S = 3/2) per unit cell aligns in the same out-of-plane direction (Fig. 1a). The minimum model to describe this ferromagnetism in the monolayer CrI_3_ is the Ising spin Hamiltonian, $H = - \frac{1}{2}\mathop {\sum }\limits_{\langle i,j \rangle} \left( {J_{{\mathrm{XY}}}\left( {S_i^{\mathrm{X}}S_j^{\mathrm{X}} + S_i^{\mathrm{Y}}S_j^{\mathrm{Y}}} \right) + J_{\mathrm{Z}}S_i^{\mathrm{Z}}S_j^{\mathrm{Z}}} \right)$, where $S_{i(j)}^{{\mathrm{X}}({\mathrm{Y}},{\mathrm{Z}})}$ is the spin operator along the X (Y, Z) direction at the Cr^3+^ site *i* (*j*); *J*_Z_ and *J*_XY_ are the exchange coupling constants for the out-of-plane and the in-plane spin components, respectively, and satisfy *J*_Z_>*J*_XY_> 0 for Ising ferromagnetism; and 〈*i*,*j*〉 denotes the approximation of the nearest-neighbor exchange coupling. Figure 1b shows the calculated spin wave dispersion relation along the high-symmetry directions (*Κ–Γ–Μ–Κ*) of the first Brillouin zone (Fig. 1b, inset). Because there are two magnetic Cr^3+^ cations per primitive cell, there are two spin wave branches whose eigenstates contain in-phase (lower branch) and out-of-phase (upper branch) spin precessions between the two sublattices^26^. Of particular interest to Raman scattering, as well as the tunneling geometry of the magnetic filter devices, are the spin wave states at the Brillouin zone center (*Γ* point, zero-momentum) describing the uniform precession of the spins about the easy axis. The energy barrier from the ground state to the lower branch is proportional to the magnetic anisotropy, $\Delta _{\mathrm{L}} = \frac{9}{4}\left( {J_{\mathrm{Z}} - J_{{\mathrm{XY}}}} \right)$, which is the energy cost for the spins to uniformly tilt off the easy axis in this excited state (Fig. 1d). The energy barrier for the upper branch results from a combined effect of both the magnetic anisotropy and the in-plane exchange coupling, $\Delta _{\mathrm{U}} = \frac{9}{4}\left( {J_{\mathrm{Z}} + J_{{\mathrm{XY}}}} \right)$, corresponding to the energy needed for tilting the spins at the two Cr^3+^ sublattices in the opposite directions upon this excitation (Fig. 1c).

### Raman signature of two sets of zero-momentum spin waves

To study both zero-momentum spin waves in 2D CrI_3_ crystals, we fabricated CrI_3_ thin flakes fully encapsulated by hexagonal boron nitride (hBN) (see sample fabrication details in Methods and thickness characterization in Supplementary Note 1) and performed micro-Raman spectroscopy measurements on them as a function of layer number and temperature, as frequency-resolved magnetic Raman scattering cross section is directly proportional to the time-domain magnetic correlation function^27^. In contrast to phonon Raman scattering which preserves time-reversal symmetry (TRS) and thus has symmetric Raman tensors^28^, the leading-order one-magnon Raman scattering involves spin flipping (ΔS = ±1) that breaks TRS, and consequently corresponds to antisymmetric Raman tensors^29,30^. Based on the difference in the Raman tensors, we can therefore readily distinguish magnon Raman modes from phonon modes via polarization selection rules. In our Raman measurements with the backscattering geometry, the polarizations of the incident and the scattered light were kept to be either parallel or perpendicular to each other, and could be rotated together with respect to the in-plane crystal axis by any arbitrary angle *ϕ*. The incident photon energy of 1.96 eV was chosen to be on resonance with the charge transfer and the Cr^3+ 4^*A*_2_ to ^4^*T*_1_ transitions of CrI_3_ in order to increase the Raman sensitivity of magnon scattering^4^ (see Supplementary Note 2 for a comparison with the nonresonant Raman spectra).

**Fig. 2:**
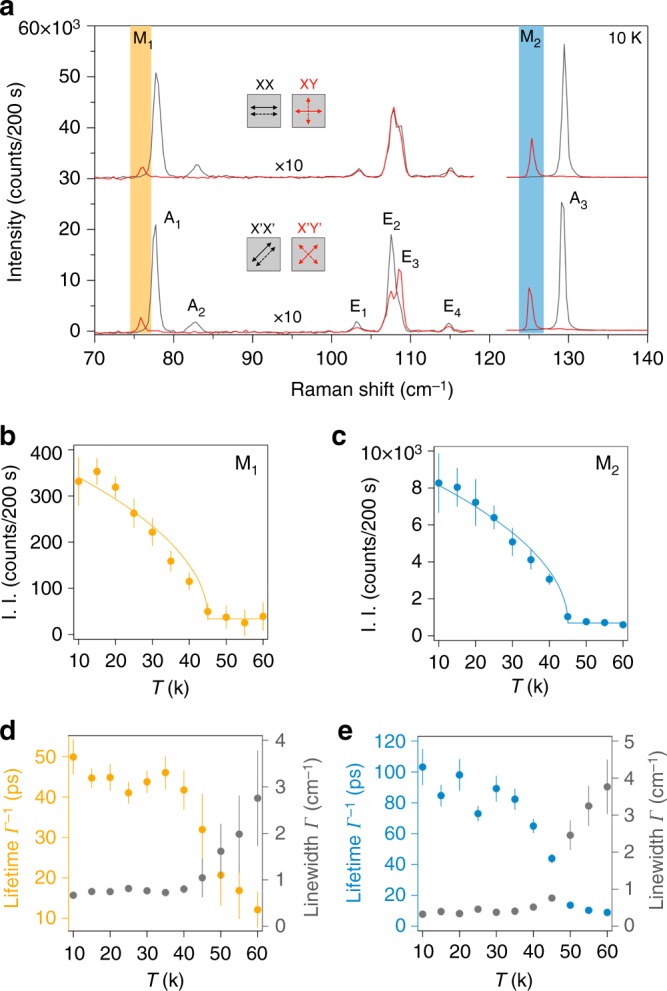
Detection of the zero-momentum magnons in thick CrI_3_. **a** Raman spectra of a thick CrI_3_ flake (13 layers) at low temperature (10 K) in the parallel and cross-channels at *ϕ* = 0^o^ (XX and XY) and at *ϕ* = 45^o^ (X’X’ and X’Y’). Magnon modes, M_1_ and M_2_, appearing only in the cross-channels (XY and X’Y’), are highlighted in yellow and blue. Phonon modes are labeled as A_1_, A_2_, E_1_–E_4_, and A_3_. The spectral intensities in the 70–120 cm^–1^ range are multiplied by a factor of 10. The spectra in the XX and XY channels are vertically offset for clarity. The spectra are acquired using a 633-nm excitation laser. **b**, **c** Temperature dependence of I. I. of the M_1_ and M_2_ magnon modes, respectively. Solid curves are fits to $I_0 + I\sqrt {T_{\mathrm{C}} - T}$.** d**, **e** Temperature dependence of the lifetime (*Γ*^−1^, left axis) and the linewidth (*Γ*, right axis) of the M_1_ and M_2_ magnon modes, respectively. Error bars in **b**–**e** represent the two standard errors of fitting parameters in the Lorentzian fits to individual temperature-dependent Raman spectra

Figure 2a shows low-temperature (10 K) Raman spectra taken on a 13-layer (13 L) CrI_3_ flake in both parallel and cross-polarization selection channels at *ϕ* = 0^o^ and *ϕ* = 45^o^, denoted as XX, XY, X’X’, and X’Y’, respectively. In total, there are nine Raman-active modes observed, which can be categorized into three groups based on their selection rules. First and most remarkably, the M_1_ and M_2_ modes are only present in the cross-channels (XY and X’Y’) and are absent in the parallel channels (XX and X’X’) within our detection resolution. This leads to the unique identification of purely antisymmetric Raman tensors for these two modes, evidence of them arising from the two zero-momentum magnons depicted in Fig. 1c and 1d. Of equal interest are their high frequencies at 76 cm^–1^ (2.28 THz, or 9.4 meV) and 125 cm^–1^ (3.75 THz, or 15.5 meV), respectively, which are three orders of magnitude higher than those of the conventional ferromagnets used in most spintronic devices today (in the GHz range)^19,20^. Even though our measured magnon frequencies are in a similar energy scale as those reported in Ref. 11 (3 and 7 meV, and possibly 17 meV), their quantitative difference is significant and invites further investigations on the spin dynamics in 2D CrI_3_. Furthermore, by substituting Δ_L_ and Δ_U_ with the two magnon frequencies above, the exchange coupling constants for the in-plane (*J*_XY_) and the out-of-plane (*J*_Z_) spins are determined to be 11 cm^–1^ and 44 cm^–1^, respectively, in good agreement with that obtained from generalized calculations of magnetic coupling constants for bulk CrI_3_^31^. Second, the A_1_, A_2_, and A_3_ modes show up only in the parallel channels (XX and X’X’) without any *ϕ* dependence, and therefore these are the *A*_g_ phonon modes under the rhombohedral crystal point group *C*_3i_ (space group $R\overline 3$)^32^. Third, the E_1_, E_2_, E_3_, and E_4_ modes are the *E*_g_ phonon modes of *C*_3i_, because of their appearance in both parallel and cross-channels, as well as the rotational anisotropy of their intensities (see detailed analysis for all Raman modes in Supplementary Note 3).

### Temperature dependence of spin waves

Having identified the two single-magnon modes in the Raman spectra at low temperature, we proceed to evaluate their temperature dependence. To see the results quantitatively, we fit the magnon modes with a Lorentzian function of the form $\frac{{A(\varGamma /2)^2}}{{(\omega - \omega _{\mathrm{o}})^2 + (\varGamma /2)^2}}$, where *ω*_o_, *Γ*, and *A* are the central frequency, linewidth, and peak intensity of the magnon mode, respectively. Figure 2b, c show the temperature dependence of the I. I., $\frac{{\mathrm{\pi }}}{2}A\varGamma$, of the two magnon modes. Clearly, both traces exhibit a clear upturn below a critical temperature *T*_C_ = 45 K (the same value for bulk CrI_3_), that is, however, lower than the bulk Curie temperature of 60 K determined by the magnetic susceptibility measurements under a magnetic field of 0.1 T (see the magnetic susceptibility data and Raman data on bulk CrI_3_ in Supplementary Note 4). The temperature dependence of the magnon lifetimes (inverse of the linewidths, *Γ*^−1^, Fig. 2d, e), on the other hand, shows that a short lifetime of < 10 ps sets in at 60 K and saturates at about 50 ps (100 ps) around 45 K for the M_1_ (M_2_) magnon. This, together with the divergent behavior of the linewidth temperature dependence (see Fig. 2d, e), indicates that strong magnetic fluctuations are present before the static magnetic order is established at 45 K. It is therefore likely that 60 K marks the onset of the field-stabilized magnetic correlations, while 45 K denotes the intrinsic transition to the spontaneous ferromagnetism, reconciling the difference between the critical temperatures measured by the two different experimental techniques. This is also consistent with the temperature-dependent magneto-optical Kerr effect^2^ and tunneling^7,12^ measurements of thin samples at zero fields, and explains the difference between the tunneling resistance with^11^ and without^7,12^ a magnetic field appearing above *T*_C_.

### Thickness dependence of spin waves

**Fig. 3:**
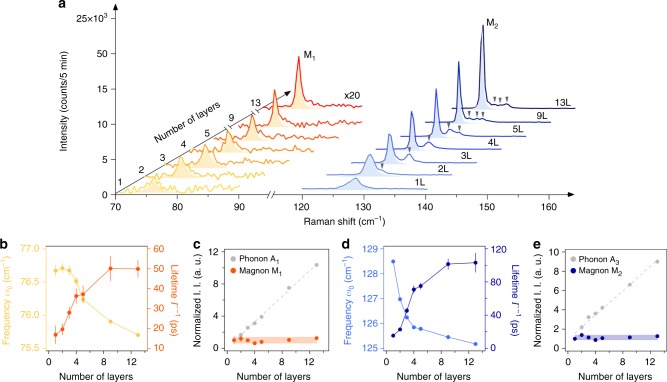
Layer number dependence of the zero-momentum magnon characteristics. **a** Raman spectra of the M_1_ (intensity multiplied by 20) and M_2_ magnons for 1–5 L, 9 L, and 13 L samples. Light yellow and blue shaded areas are the Lorentzian fits for the M_1_ and M_2_ magnon modes, respectively. The gray triangles highlight the satellite magnon peaks. **b** Frequency (*ω*_0_, left axis) and lifetime (*Γ*^*–1*^, right axis) of the M_1_ magnon as a function of layer number. **c** I. I. of the M_1_ magnon as a function of layer number with I. I. of the A_1_ phonon shown in gray for comparison. **d**, **e** Plots for M_2_ that are similar to **b**, **c**, with I. I. of the A_3_ phonon plotted in gray in **e** for comparison. Error bars in **b**–**e** represent the two SEs of fitting parameters in the Lorentzian fits to individual Raman spectra at different thicknesses

To investigate how thermal fluctuations impact the intrinsic 2D ferromagnetism and its excitations, we performed a systematic Raman study of the M_1_ and M_2_ magnon modes measured on atomically thin CrI_3_ crystals ranging from 13 L to monolayer (1 L). It is clear from the data taken at 10 K in Fig. 3a that both main magnon modes (M_1_ and M_2_) and their satellite modes (highlighted with gray triangles in Fig. 3a) have a notable layer number dependence. First, the satellite magnon modes arise from the finite thickness effect in thin layers, in which broken translational symmetry perpendicular to the basal plane makes single-magnon modes with finite out-of-plane momenta accessible in Raman scattering^33,34^ (see the same effect for phonons in Supplementary Note 5). The small energy separation between the main mode and the nearest satellite, on the order of 3 cm^–1^, indicates the weak interlayer magnetic coupling strength^26^, consistent with the small training magnetic field of less than 1 T reported in literature^2,8,11^. Moreover, as the layer number decreases, there are fewer but stronger observable satellite magnon modes in the Raman spectra. Second, the two main magnon modes, M_1_ and M_2_, persist down to the monolayer with symmetric lineshapes, while their peak intensities drop and their linewidths broaden with decreasing layer numbers.

To understand the layer number dependence of the M_1_ and M_2_ magnons in greater detail, we extracted the magnon mode frequencies (*ω*_o_), lifetimes (*Γ*^−1^), and I. I. ($\frac{{\mathrm{\pi }}}{2}A\varGamma$), from fitting their Raman spectra with the Lorentz function ($\frac{{A(\varGamma /2)^2}}{{(\omega - \omega _{\mathrm{o}})^2 + (\varGamma /2)^2}}$), and the results are summarized in Fig. 3b–e. The frequencies of the M_1_ and M_2_ magnons increase slightly, by about 0.8 cm^–1^ (1.1%) and 3 cm^–1^ (2.4%), respectively, as the layer number decreases from 13 L to 1 L, possibly because the reduced electronic screening in thinner samples enhances the exchange coupling. In sharp contrast, their lifetimes drop significantly, from about 50 ps (100 ps) in 13 L to 15 ps in 1 L for M_1_ (M_2_), which is consistent with the increased thermal fluctuations in 2D. Despite this decrease, even in the monolayer, the lifetimes are still more than one order of magnitude larger than the corresponding magnon temporal periods, about 30 times for M_1_ and 50 times for M_2_. This ratio of magnon lifetime to the temporal period in 2D CrI_3_ is significantly higher than that of the Heisenberg ferromagnets^19,20^ and at least comparable to, if not greater than, that of the antiferromagnets^20–22^, making coherent control of both THz spin waves in the time domain feasible down to the monolayer limit of CrI_3_. Remarkably, I. I. of both magnons, which is known to be proportional to the magnon density, remain nearly constant and independent of the layer number, while that of the phonons scale linearly with the thickness (Fig. 3c, e). This observation on the M_1_ and M_2_ magnons is consistent with surface magnons whose density is thickness-independent. Considering that surface magnons have been theoretically predicted in 2D honeycomb ferromagnets^26^, although mainly at different wave vectors *K* points, it might not be unreasonable to speculate the surface origin of the two THz magnon modes that we have detected here.

### Phase diagram of 2D CrI_3_

**Fig. 4:**
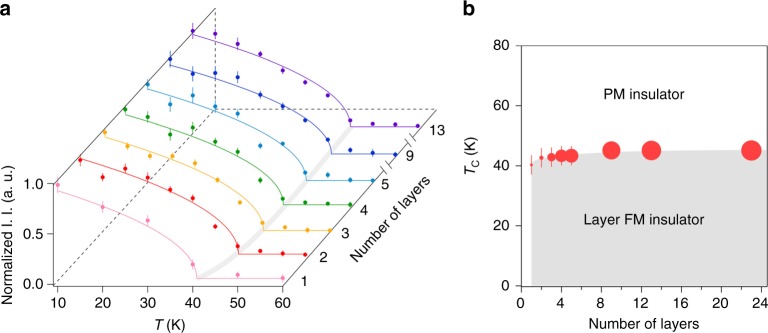
Temperature versus layer number phase diagram of the 2D layer ferromagnetic CrI_3_. **a** Temperature dependence of I. I. of the M_2_ magnon normalized to the value at 10 K as a function of layer number. The colored solid curves are fits to $I_0 + I\sqrt {T_{\mathrm{C}} - T}$. The gray curve is the guide to the eye of the evolution of *T*_C_. Error bars correspond to the two SEs of normalized I. I. in the Lorentzian fits at individual temperatures for different thicknesses of 2D CrI_3_. **b** Temperature versus layer number phase diagram (PM for paramagnetism and FM for ferromagnetism). The size of the data points represents the M_2_ magnon lifetime. Error bars correspond to the two SEs of *T*_C_ in the fits in **a**

By carrying out temperature-dependent Raman measurements and analysis similar to Fig. 2b, c for different thickness samples, we tracked the layer dependence of the 2D ferromagnetism onset temperature. Figure 4a displays the temperature-dependent traces of the normalized I. I. of the M_2_ magnon plotted as a function of layer number, with the onset temperature (*T*_C_) for each trace determined by fitting with an order-parameter-like function for a ferromagnet ${\mathrm{I}}.{\mathrm{I}}. \propto \sqrt {T_{\mathrm{C}} - T}$ (see the similar plot of M_1_ in Supplementary Note 6). As the layer number decreases from 13 L to 1 L, the extracted *T*_C_ has an observable decline from 45 K to 40 K. This approximately 12% suppression in *T*_C_ is in sharp contrast to the slight enhancement of the magnon frequencies, i.e., 1.1% increase of Δ_L_ for M_1_ and 2.4% of Δ_U_ for M_2_, which then suggests that the drop in *T*_C_ is due to stronger thermal fluctuations in thinner samples^35^. Nevertheless, the finite *T*_C_ for all samples with various thicknesses establishes a phase boundary for the intrinsic transition to the intralayer ferromagnetism in 2D CrI_3_ (see Fig. 4b).

## Discussion

We have identified two branches of THz spin waves with their lifetime on the order of 10–100 ps in a 2D Ising ferromagnet, CrI_3_, whose magnetic onset temperature *T*_C_ remains close to that of their bulk crystal. The robust THz magnons in 2D CrI_3_ are in stark contrast to spin waves in conventional metallic ferromagnetic thin films that occur at relatively low, GHz frequencies^16^ and also show significant substrate-dependence^36–38^. Similar to many antiferromagnets, 2D CrI_3_ is a semiconductor that possesses high-frequency, long-lived spin waves and is free of stray magnetic fields within 2D domains^39,40^. Different from bulk antiferromagnets, 2D CrI_3_ couples efficiently with external magnetic fields^2–4,8,11,12^ and can be tailored in various device geometries with definitive thicknesses^6,10^. We envision that these unique characteristics of spin waves in 2D CrI_3_ will provide unprecedented opportunities for applications in ultrafast and ultracompact spintronic devices.

## Methods

### Magnon dispersion calculations

The Ising Hamiltonian with anisotropic exchange coupling is transformed by applying the Holstein–Primakoff transformation. The single-site spin operators, $S_\phi ^ +$ and $S_\phi ^ -$, are related to the momentum space magnon creation and annihilation operators, $\alpha _{\mathbf{k}}^\dagger$ and *α*_***k***_, as $S_\phi ^ + = S_\phi ^{\mathrm{X}} + {\mathrm{i}}S_\phi ^{\mathrm{Y}} = \sqrt {2S/N} \mathop {\sum }\limits_{\mathbf{k}} \alpha _{\mathbf{k}}e^{{\mathrm{i}}{\mathbf{k}} \cdot {\mathbf{r}}_\phi }$, $S_\phi ^ - = S_\phi ^{\mathrm{X}} - {\mathrm{i}}S_\phi ^{\mathrm{Y}} = \sqrt {2S/N} \mathop {\sum }\limits_{\mathbf{k}} \alpha _{\mathbf{k}}^\dagger e^{ - {\mathrm{i}}{\mathbf{k}} \cdot {\mathbf{r}}_\phi }$, and $S_\phi ^{\mathrm{Z}} = S - 1/N\mathop {\sum}\limits_{{\mathbf{k}},{\mathbf{k}}^\prime } {\alpha _{\mathbf{k}}^\dagger \alpha _{{\mathbf{k}}^\prime }e^{{\mathrm{i}}({\mathbf{k}}^\prime - {\mathbf{k}}) \cdot {\mathbf{r}}_\phi }}$ where *ϕ* = a or b, corresponding to the two Cr^3+^ sublattices (*n* = 2 and *S* = 3/2). The bosonic Hamiltonian is then diagonalized using wavefunction $$\psi _{\mathbf{k}} = \left( {\begin{array}{*{20}{c}} {a_{\mathbf{k}}} \\ {b_{\mathbf{k}}} \end{array}} \right)$$ to extract the magnon dispersion relations and the eigenvectors.

### Growth of CrI_3_ single crystals

The single crystals of CrI_3_ were grown by the chemical vapor transport method. Chromium powder (99.99% purity) and iodine flakes (99.999%) in a 1:3 molar ratio were put into a silicon tube with a length of 200 mm and an inner diameter of 14 mm. The tube was pumped down to 0.01 Pa and sealed under vacuum, and then placed in a two-zone horizontal tube furnace. The two growth zones were raised up slowly to 903 K and 823 K for 2 days, and were then held there for another 7 days. Shiny, black, plate-like crystals with lateral dimensions of up to several millimeters can be obtained from the growth. In order to avoid degradation, the CrI_3_ crystals were stored in a glovebox filled with nitrogen.

### Fabrication of few-layer samples

CrI_3_ samples were exfoliated in a nitrogen-filled glovebox and the thickness of the flakes was first estimated by the optical contrast. Using a polymer-stamping technique inside the glove box, CrI_3_ flakes were sandwiched between two few-layer hBN flakes to avoid surface reaction with oxygen and moisture in the ambient environment. The encapsulated CrI_3_ samples were then moved out of the glove box for Raman spectroscopy measurements. After Raman spectroscopy measurements, the thicknesses of the encapsulated CrI_3_ flakes were determined by the atomic force microscopy (AFM) measurements.

### Raman spectroscopy

Raman spectroscopy measurements were carried out using both a 633-nm and a 532-nm excitation laser with a beam spot size of ~3 μm. The laser power was kept at 80 μW, corresponding to a similar fluence used in literature (10 μW over an ~1-μm-diameter area), to minimize the local heating effect. Backscattering geometry was used. The scattered light was dispersed by a Horiba Labram HR Raman spectrometer and detected by a thermoelectric cooled CCD camera. Selection rule channels XX and XY denote the parallel and cross-polarizations of incident and scattered light at *ϕ* = 0^o^; X’X’ and X’Y’ represent the parallel and cross-channels at *ϕ* = 45^o^. A closed-cycle helium cryostat was interfaced with the micro-Raman system for the temperature-dependent measurements. All thermal cycles were performed at a base pressure lower than 7 × 10^–7^ Torr.

## Electronic supplementary material

Supplementary Information

Source Data Figure 2b-e

Source Data Figure 3b-e (1L data)

Source Data Figure 3b-e (2L data)

Source Data Figure 3b-e (3L data)

Source Data Figure 3b-e (4L data)

Source Data Figure 3b-e (5L data)

Source Data Figure 3b-e (9L data)

Source Data Figure 3b-e (13L data)

Source Data Figure 4a (1L data)

Source Data Figure 4a (2L data)

Source Data Figure 4a (3L data)

Source Data Figure 4a (4L data)

Source Data Figure 4a (5L data)

Source Data Figure 4a (9L data)

Source Data Figure 4a (13L data)

Source Data Supplementary Figure 5

Source Data Supplementary Figure 6

Source Data Supplementary Figure 8 (1L data)

Source Data Supplementary Figure 8 (2L data)

Source Data Supplementary Figure 8 (3L data)

Source Data Supplementary Figure 8 (4L data)

Source Data Supplementary Figure 8 (5L data)

Source Data Supplementary Figure 8 (9L data)

Source Data Supplementary Figure 8 (13L data)

## Data Availability

The data that support the plots of this paper are available from the corresponding authors upon reasonable request. And the source data underlying Figs. 2b–e, 3b–e, and 4a, b and Supplementary Figs 5b–g, 6b–g, and 8 are provided as a Source Data file.
