# Supplementary material for: Raman fingerprint of two terahertz spin wave branches in a two-dimensional honeycomb Ising ferromagnet
Source: Nat Commun. 2018 Nov 30;9:5122. doi: 10.1038/s41467-018-07547-6 (PMC6269484; doi:10.1038/s41467-018-07547-6)
Supplement: Supplementary file 1 — Supplementary Information [file 41467_2018_7547_MOESM1_ESM.pdf]

Raman Fingerprint of Two Terahertz Spin Wave Branches in A Two-Dimensional Honeycomb Ising Ferromagnet

Supplementary Information

Jin *et al*

Supplementary Note 1: Thickness characterization of CrI_3 thin layers

Supplementary Figure 1 shows the characterization of the thickness of a representative CrI_3 flake on which the Raman spectra of 2-5L CrI_3 shown in the main text were acquired. As stated in the Methods section, the thickness of the sample was first estimated by the optical contrast (supplementary Figure 1a and b), and then fully encapsulated by hBN flakes (supplementary Figure 1c). To prevent potential damage, the height profiles for the various thicknesses were measured by ambient atomic force microscopy (AFM) after Raman spectroscopy measurements. As a monolayer CrI_3 film between two hBN flakes was determined to be 0.7 nm, this flake contains regions with four different thicknesses, including 1.4 nm (2L), 2.1 nm (3L), 2.8 nm (4L) and 3.6 nm (5L) as shown in supplementary Figure 1d-g.

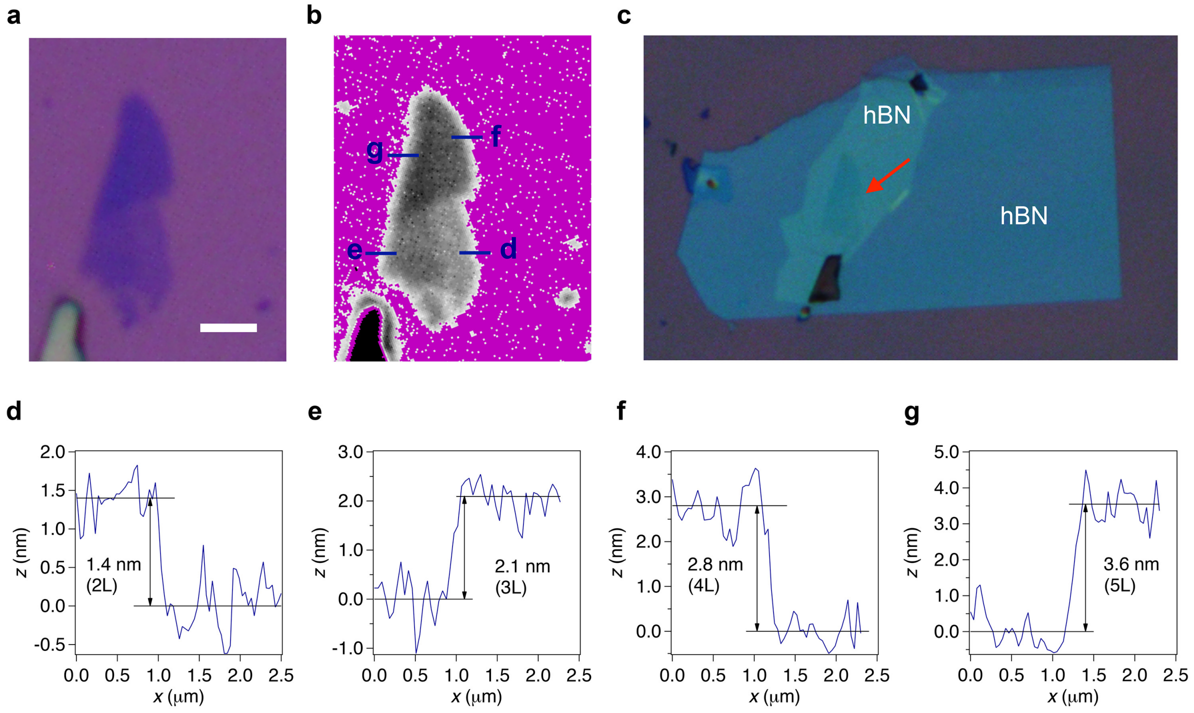

Supplementary Figure 1. Thickness characterization of CrI_3 flakes. **a**, Optical microscope image of a representative CrI_3 flake. Scale bar is 5 μm. **b**, Pseudocolor map of the same flake for a better visualization of the optical contrast. **c**, Optical microscope image of the same flake (marked by the red arrow) sandwiched between two few-layer hBN flakes. **d-g**, AFM line cuts across the flake edges marked in **b** with the corresponding thicknesses and layer numbers labeled.

Supplementary Note 2: Comparison between on and off-resonance Raman spectra

Raman spectra from the 13L CrI_3, acquired using both 633 nm (on-resonance) and 532 nm (off-resonance) excitation lasers under similar measurement conditions, are shown in supplementary Figure 2. Comparing to the off-resonance Raman spectra, the resonant Raman intensities of the M_2 magnon mode and the A_3 phonon mode are significantly enhanced by a factor of $\sim$20 while the rest modes are enhanced by $\sim$5. Note that the M_1 and M_2 magnon modes appear in the off-resonance spectra with the identical selection rules as in the resonant spectra, which rules out the possibility that the M_1 and M_2 magnon modes are resonance-induced symmetry forbidden phonon modes. A new phonon mode (labeled as E_4. in Fig. 2 of the main text) appears at $\sim$115 cm^{-1} in the resonant spectra. A detailed analysis of E_4 mode is beyond the scope of this paper and will be discussed in a separate work.

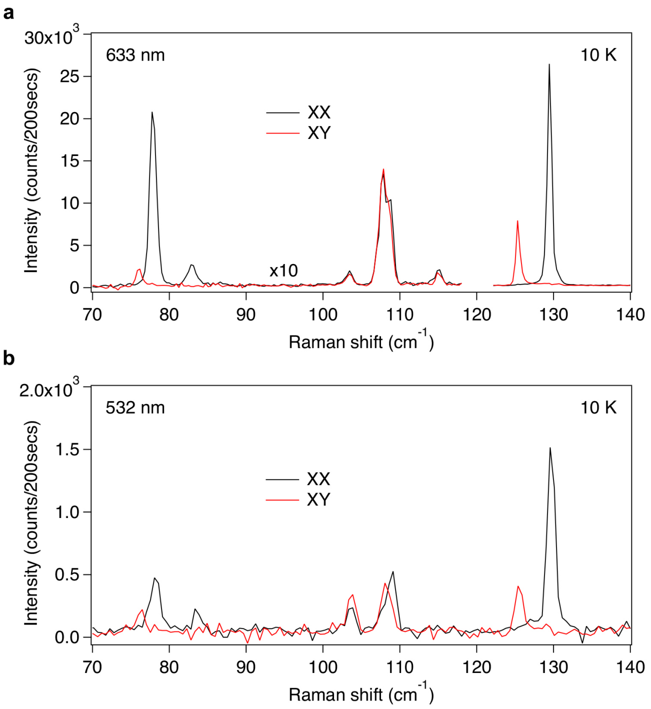

Supplementary Figure 2. Comparison between on and off-resonance Raman spectra. Raman spectra from a 13L CrI_3 flake in the XX and XY channels acquired at low temperature (10 K) using **a**, a 633 nm and **b**, a 532 nm excitation laser.

Supplementary Note 3: Symmetry analysis on Raman active phonons

At low temperature ($T < 240$ K), CrI_3 crystal has a Rhombohedral structure (point group $C_{3\mathrm{i}}$ and space group $R\bar{3}$). A factor group analysis reveals that 21 optical modes are expected with irreducible representation $\Gamma_{\mathrm{optical}} = 4A_{\mathrm{g}} + 4E_{\mathrm{g}} + 3A_{\mathrm{u}} + 3E_{\mathrm{u}}$. Among them, A_{g} and E_{g} modes are Raman active, whose Raman tensors are given by

$$\chi(A_{\mathrm{g}}) = \begin{pmatrix} a & 0 & 0 \\ 0 & a & 0 \\ 0 & 0 & b \end{pmatrix}, \qquad \chi(E_{\mathrm{g}}) = \begin{pmatrix} c & d & e \\ d & -c & f \\ e & f & 0 \end{pmatrix}$$

The Raman intensity is $I \propto |\langle E_i|\chi|E_s\rangle|^2$, where E_{i} and E_{s} are the electric field of the incident and scattered light, respectively. In the backscattering geometry, the angular dependence of the Raman intensity in the parallel and cross channels is shown in supplementary Figure 3. Here, A_{g} modes only appear in the parallel channel with isotropic Raman intensities, while E_{g} modes can be observed in both parallel and cross channels with anisotropic Raman intensities. Therefore, by comparing the selection rules of the measured Raman spectra with the symmetry analysis above, we can unambiguously assign A_1, A_2 and A_3 mode to be A_{g} modes, and E_1-E_4 modes to E_{g} modes.

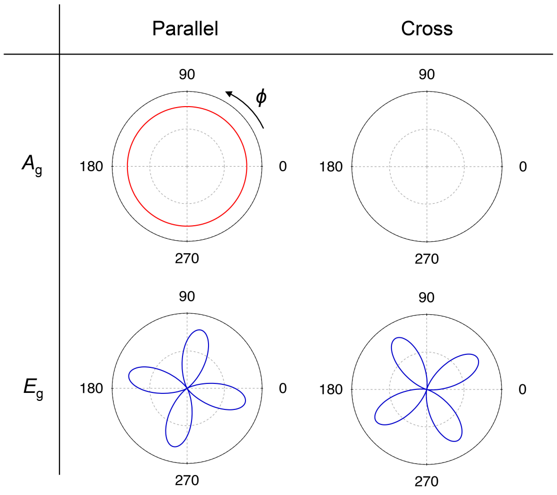

Supplementary Figure 3. Simulated angular dependence of the Raman intensities. Polar plots of angular dependent Raman intensities for A_{g} and E_{g} modes in the parallel and cross channels. The polarization angle with respect to the horizontal axis is denoted as ϕ.

Supplementary Note 4: Magnetization and Raman data from bulk CrI_3

Out-of-plane and in-plane magnetization measurements on CrI_3 single crystals were performed with the applied magnetic fields of 0.1 T, 0.5 T and 5 T. The temperature dependence of the magnetization clearly exhibits the ferromagnetic nature of CrI_3, and the Curie temperature of 60 K is determined from the lowest field (0.1 T, supplementary Figure 4a) data. The magnetization becomes nearly isotropic at a magnetic field as high as 5 T (supplementary Figure 4c), while significant magnetic anisotropy is observed below Curie temperature at lower fields (0.1 and 0.5 T, supplementary Figure 4a-b).

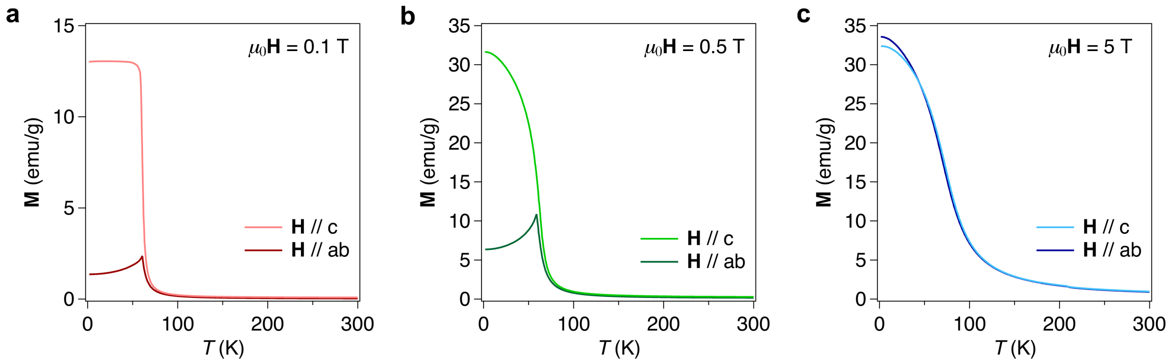

Supplementary Figure 4. Magnetization data from bulk CrI_3 crystals. Out-of-plane (**H** // c) and in-plane (**H** // ab) magnetization as a function of temperature measured with various applied magnetic fields (μ_0**H**) **a**, 0.1 T, **b**, 0.5 T and **c**, 5 T.

We further performed Raman spectroscopy measurements on a freshly cleaved CrI_3 bulk and a 75L thick CrI_3 flake that is prepared the same way as other flakes. The result is shown in supplementary Figure 5 and 6, and summarized as follows. Both bulk and 75L CrI_3 have very similar results as 13L CrI_3, including similar magnon frequency and linewidth, and similar temperature dependence and magnetic onset temperature. This indicate that the 13L CrI_3 is thick enough to represent the bulk-like magnetic properties, and therefore we choose to use 13L CrI_3 data in the main text for a consistent comparison with other CrI_3 thin layers prepared in the same way.

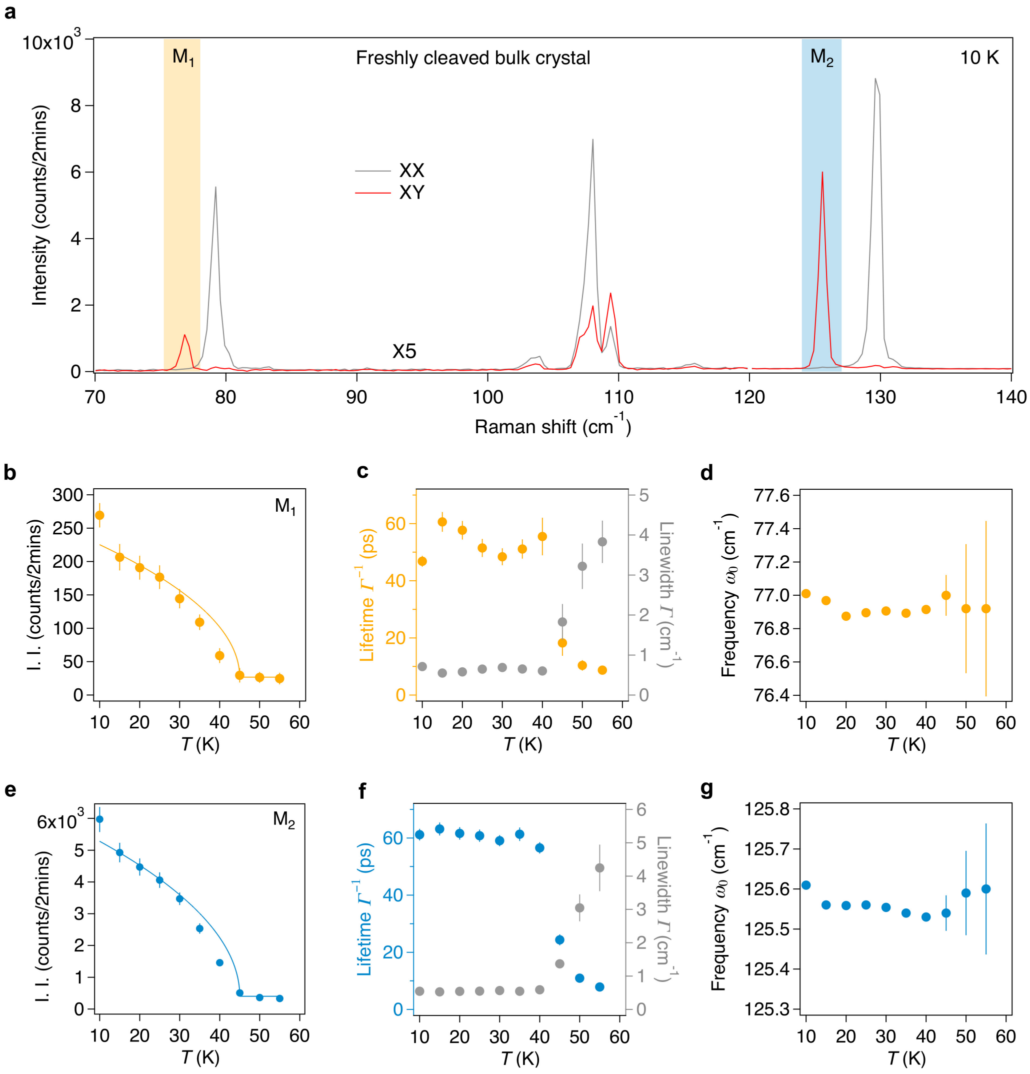

Supplementary Figure 5. Raman data taken on freshly cleaved bulk $\mathbf{CrI_3}$. **a**, Raman spectra of a freshly cleaved bulk CrI_3 crystal at low temperature (10 K) in the parallel and cross channels at $\phi = 0°$ (XX and XY). Magnon modes, M_1 and M_2, appearing only in the cross channels (XY) are highlighted in yellow and blue. The spectral intensities in the 70-120 cm^{-1} range are multiplied by a factor of 5. The spectra are acquired using a 633 nm excitation laser. **b-d**, Temperature dependence of I. I., lifetime ($\mathit{\Gamma}^{-1}$, left axis) and the linewidth ($\mathit{\Gamma}$, right axis), and frequency of the M_1 magnon mode, respectively. Solid curve in **b** is fit to $I_0 + I\sqrt{T_C - T}$. **e-g**, Same plots for M_2 as in **b-d**. Error bars represent the two standard errors of fitting parameters in the Lorentzian fits to individual Raman spectra at different temperatures.

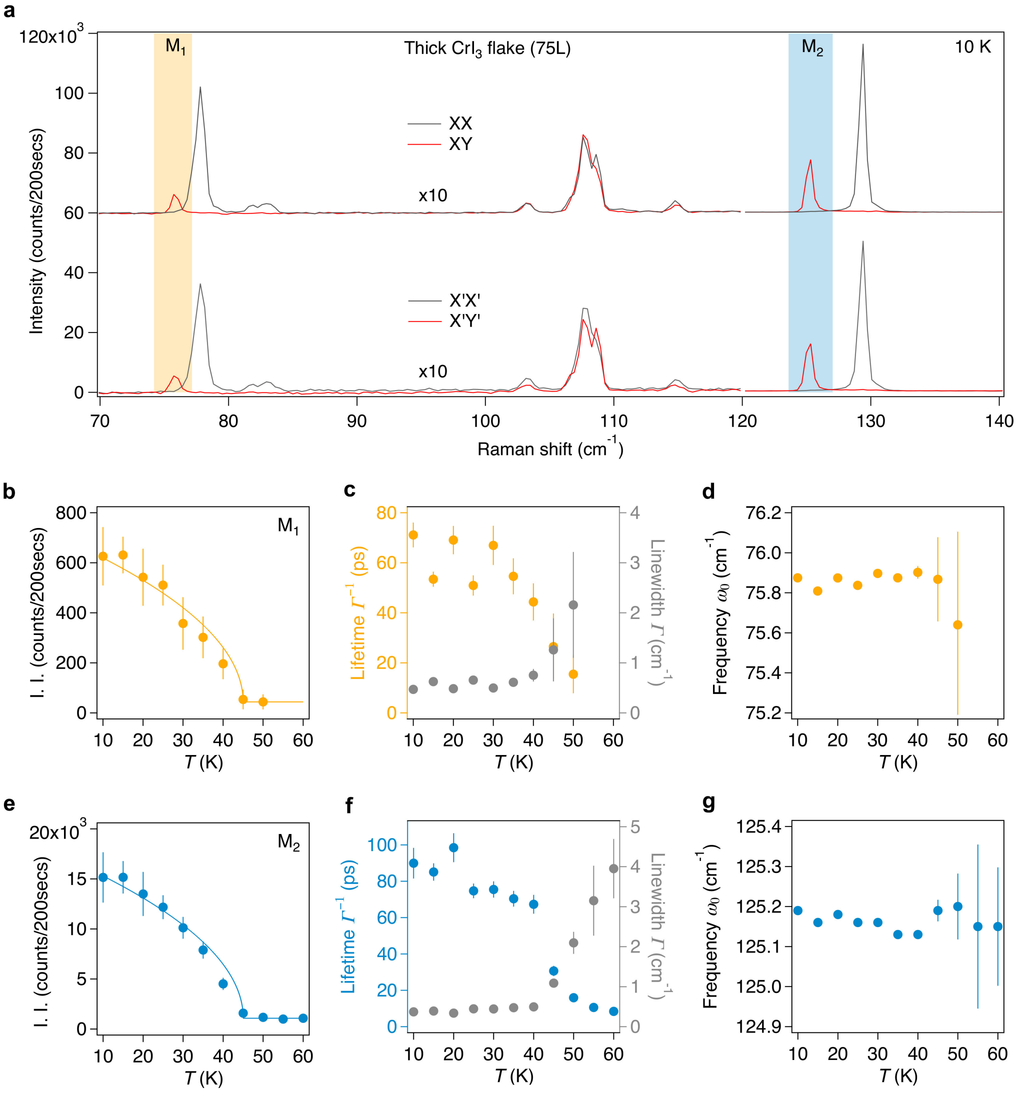

Supplementary Figure 6. Raman data taken on a 75L-thick CrI_3 flake. **a**, Raman spectra of a thick CrI_3 flake (75L) at low temperature (10 K) in the parallel and cross channels at $\phi = 0°$ (XX and XY) and at $\phi = 45°$ (X′X′ and X′Y′). Magnon modes, M_1 and M_2, appearing only in the cross channels (XY and X′Y′) are highlighted in yellow and blue. The spectral intensities in the 70-120 cm^{-1} range are multiplied by a factor of 10. The spectra are acquired using a 633 nm excitation laser. **b-d**, Temperature dependence of I. I., lifetime ($\mathit{\Gamma}^{-1}$, left axis) and the linewidth ($\mathit{\Gamma}$, right axis), and frequency of the M_1 magnon mode, respectively. Solid curve in **b** is fit to $I_0 + I\sqrt{T_C - T}$. **e-g**, Same plots for M_2 as in **b-d**. Error bars represent the two standard errors of fitting parameters in the Lorentzian fits to individual Raman spectra at different temperatures.

Supplementary Note 5: Satellite phonon modes arising from the finite thickness effect

Supplementary Figure 7 shows the A_3 phonon mode from 1-5L, 9L and 13L samples acquired in the parallel channel at 10 K. Satellite peaks appear at the lower frequency side of the main peak. Note that these satellite phonon peaks are at different frequencies from that of the M_2 magnon, which rules out the possibility of the polarization leakage from the cross channel. Similar to the satellite peaks of the M_2 magnon, the satellite peaks here come from the finite thickness effect, which are less prominent in thick sample (13L) and absent in the monolayer.

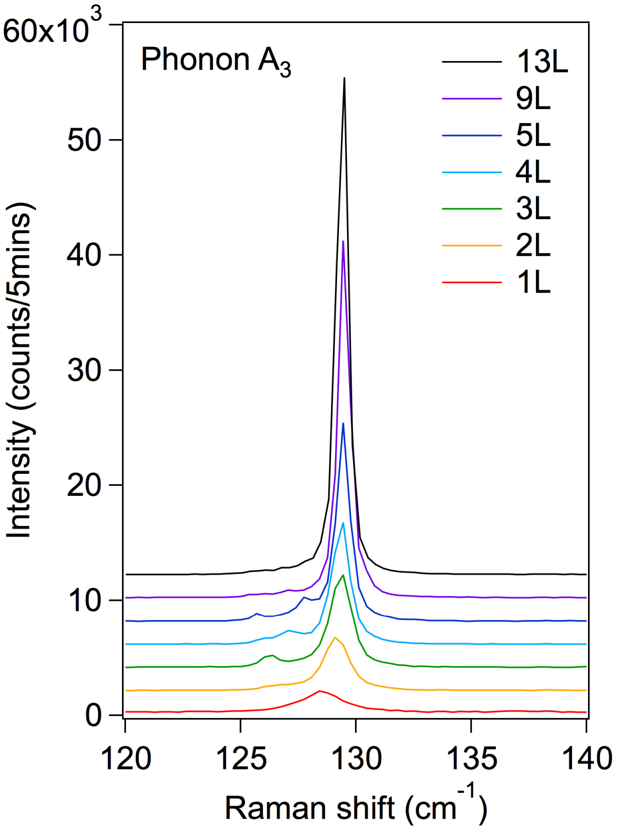

Supplementary Figure 7. Satellite phonon modes in CrI_3 flakes with varying thicknesses. The A_3 phonon mode for 1-5L, 9L and 13L samples acquired in the parallel channel at 10 K. Spectra are vertically offset for clarity.

Supplementary Note 6: Temperature dependence of M_1 as a function of thickness

Supplementary Figure 8 shows the temperature dependence of I. I. of the M_1 magnon normalized to the value at 10 K as a function of layer number. Similar as the M_2 magnon, normalized I. I. exhibits an order-parameter-like behavior (I. I. $\propto \sqrt{T_C - T}$) as temperature approaches T_C from below. T_C determined from the M_1 magnon is slightly lower than that of the M_2 magnon, in particular for the monolayer sample. A potential explanation could be that the M_1 magnon is at lower energy than the M_2 magnon and therefore is more affected by the increased thermal fluctuations in 2D.

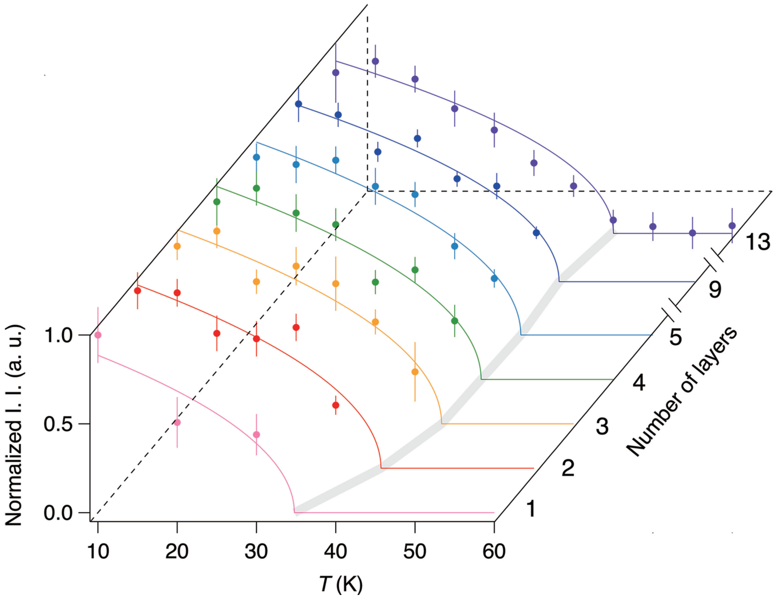

Supplementary Figure 8. Temperature dependence of M_1 as a function of layer numbers. Normalized I. I of the M_1 magnon as a function of temperature for 1-5L, 9L and 13L thick samples. The gray curve is the guide to the eye of the evolution of fitted T_C. Error bars represent the two standard errors of the normalized I. I. extracted in the Lorentzian fits of temperature dependent Raman spectra with different layer numbers.
